# Sialic acid-binding lectin from bullfrog eggs inhibits human malignant mesothelioma cell growth *in vitro* and *in vivo*

**DOI:** 10.1371/journal.pone.0190653

**Published:** 2018-01-03

**Authors:** Takeo Tatsuta, Toshiyuki Satoh, Shigeki Sugawara, Akiyoshi Hara, Masahiro Hosono

**Affiliations:** 1 Division of Cell Recognition Study, Institute of Molecular Biomembrane and Glycobiology, Tohoku Medical and Pharmaceutical University, Aobaku, Sendai, Japan; 2 Department of Clinical Pharmacotherapeutics, Tohoku Medical and Pharmaceutical University, Aobaku, Sendai, Japan; Columbia University, UNITED STATES

## Abstract

Malignant mesothelioma is an aggressive cancer that results from exposure to asbestos. The therapeutic options for this type of cancer are limited; therefore, the development of novel therapeutic agents is urgently required. Sialic acid-binding lectin isolated from *Rana catesbeiana* oocytes (cSBL) is a novel therapeutic candidate for cancer, which exhibits antitumor activity mediated through RNA degradation. In the present study, we evaluated the effect of cSBL *in vitro* and *in vivo*. Xenograft-competent H2452 and MSTO human mesothelioma cell lines were treated with cSBL, and the pathway by which cSBL induces apoptosis was analyzed. *In vivo* studies were performed using nude mice inoculated with one of the two cell lines, and the effects of cSBL and pemetrexed were monitored simultaneously. Furthermore, the pharmacological interactions between the three agents (pemetrexed, cisplatin and cSBL) were statistically assessed. It was demonstrated that cSBL treatments caused morphological and biochemical apoptotic changes in both cell lines. Caspase cascade analysis revealed that an intrinsic pathway mediated cSBL-induced apoptosis. The administration of cSBL significantly inhibited tumor growth in two xenograft models, without any adverse effects. Furthermore, the combination index and dose reduction index values indicated that the cSBL + pemetrexed combination showed the highest synergism, and thus potential for reducing dosage of each drug, compared with the other combinations, including the existing pemetrexed + cisplatin regimen. cSBL exerted prominent antitumor effects on malignant mesothelioma cells *in vitro* and *in vivo*, and showed favorable effects when combined with pemetrexed. These results suggest that cSBL has potential as a novel drug for the treatment of malignant mesothelioma.

## Introduction

Malignant mesothelioma is an aggressive cancer of the mesothelial cells of serous membranes, involving the pleural and peritoneal spaces, which results from exposure to asbestos [1–3]. The mechanisms underlying the induction of DNA damage by asbestos fibers in mesothelial cells remain unclear. The production and use of asbestos is now forbidden in the majority of industrialized countries; however, it is still actively used in many developing countries [4]. As the time between asbestos exposure and disease diagnosis is >40 years on average, the incidence of mesothelioma is increasing and is projected to peak in the late 2020s, even in developed countries [5].

Malignant mesothelioma may have an epithelioid, sarcomatoid or biphasic morphology [6]. The epithelioid type is associated with a longer survival time compared with the biphasic and sarcomatoid types [7,8]. Although chemotherapeutic approaches are limited for malignant mesothelioma, it is generally accepted that patients with the epithelioid subtype respond better to treatment [9,10]. Additionally, other factors, including sex, performance status, disease stage, serum lactate dehydrogenase level, anemia and leukocytosis, reportedly influence survival in malignant mesothelioma [11]. However, it is difficult to identify the biological factors that clearly differentiate between patients with a poor prognosis and those with a more favorable prognosis, as long-term survival is rare in malignant mesothelioma [6].

There are few therapeutic options (surgery, radiation therapy, and chemotherapy) for mesothelioma [1]. The folate antimetabolite pemetrexed is a chemotherapeutic agent, which is typically used in combination with platinum-containing drugs, such as cisplatin [12,13]. This combination therapy improves the response rate, progression-free survival, overall survival, and quality of life of patients with mesothelioma [12]; however, any treatment-induced regression observed is typically transient, and local tumors rapidly relapse due to the high chemoresistance of this cancer type [4]. So far, some progresses were obtained by multimodality therapy [14]. A median survival time of up to 29 months has been reported for those who complete a trimodal therapy including chemotherapy, surgery, and hemithoracic radiation therapy [15–18]. Moreover, pleurectomy/decortication with intraoperative photodynamic therapy and adjuvant pemetrexed-based chemotherapy demonstrated 36 months median survival [19]. However, even with these aggressive approaches, the prognosis of malignant mesothelioma remains poor. Considering the predicted incidence peak and poor prognosis, as well as the fact that intrinsic and acquired resistance to existing drugs is common, further research into developing therapeutic agents for mesothelioma is essential.

There is growing interest in the use of naturally derived molecules as potential cancer therapeutics. Lectins, carbohydrate-binding proteins that occur in all organisms, are representative of such natural compounds that have great potential for cancer therapy. Among them, several sialic acid-binding lectins (SBLs), including mistletoe lectin (ML1) [20], *Maackia amurensis* seed lectin (MASL) [21], *Polygonatum cyrtonema* lectin (POL) [22] and *Haliotis discus discus* lectin (HddSBL) [23], have been reported to have antitumor effects. SBL isolated from *Rana catesbeiana* oocytes (cSBL) is a unique compound that has multifunctional activity with lectin [24,25] and ribonuclease (RNase) [26], as well as antitumor activity [25]. cSBL exerts potent cytotoxicity in various cancer cell types, but low cytotoxicity in normal cells [27]. *Rana catesbeiana* RNase (RC-RNase), an RNase purified from *R*. *catesbeiana* oocytes collected in Taiwan by Liao *et al*. is identical to cSBL [28,29]. cSBL consists of 111 amino acid residues with four disulfide bonds [29], and belongs to the vertebrate-secreted RNase family (RNase A superfamily) [30]. It has high thermal stability and strong resistance to protein denaturants [31]. These features are considered one reason for the potent antitumor activity, and provide benefits for commercialization. Furthermore, that cSBL does not associate with endogenous mammalian RNase inhibitors, and that it exerts cytotoxicity in human cancer cells via its RNase activity [25], has facilitated further research into its antitumor effects. The mechanism of cSBL-induced cytotoxicity is proposed to be as follows: cSBL binds to the cancer cell surface and is internalized. It subsequently degrades RNA in the cytosol, leading to the induction of apoptotic signaling [27]. In human leukemia Jurkat cells, cSBL was found to activate p38 and JNK mitogen-activated protein kinase (MAPK) signals and induce apoptosis via the intrinsic (mitochondrial) pathway [32]. The RNase activity was also determined to be critical for apoptosis induction in MDA-MB231 human breast cancer cells, as an amino acid-replaced mutant of cSBL that lacked RNase activity did not exhibit the apoptosis-inducing effect, even when internalized into the cells like native cSBL [33]. The efficacy of cSBL on malignant mesothelioma cells has previously been reported [34,35]; Even though cSBL hardly show cytotoxicity to normal mesothelial cell Met5A, it efficiently reduced the viability of H28 malignant mesothelioma cells, and exhibited synergistic effects with TRAIL and pemetrexed on these cells.

In our previous study, *in vivo* experiments with cSBL were performed using mice transplanted with related ascites carcinoma, Ehrlich, Mep II and Sarcoma 180 cells. cSBL prolonged their survival at non-toxic dose levels [25]. However, to date, the effect of cSBL on human malignant mesothelioma cells *in vivo* has not been elucidated. In the present study, to assess the therapeutic potential of cSBL on malignant mesothelioma, we conducted an *in vivo* study of cSBL using human malignant mesothelioma cell xenografts, and analyzed its antitumor effects on these xenograft-competent cells.

## Materials and methods

### Cell culture

The human malignant mesothelioma cell lines NCI-H2452 (H2452, #CRL-5946) and MSTO-211H (MSTO, #CRL-2081) were purchased from the American Type Cell Culture Collection (ATCC; Manassas, VA, USA). The cells were cultured in RPMI-1640 medium (Nissui Pharmaceutical Co., Tokyo, Japan) supplemented with 10% fetal bovine serum (FBS, Biosera, Nuaille, France), 100 U/mL penicillin and 100 μg/mL streptomycin (Life Technologies, Carlsbad, CA, USA) at 37°C in an atmosphere of 95% air and 5% CO_2_.

### Animals

Eggs-bearing bullfrogs (domestically caught) and 5-week-old male nude mice (BALB/c nu/nu Slc) were purchased from Japan SLC, Inc (Shizuoka, Japan). All animal experiments were carried out in accordance with the Guidelines for Animal Experiments of the Tohoku Medical and Pharmaceutical University (permission number: A16012-cn). Housing condition of the mice was kept under standard conditions approved by the institutional guidelines with free food- and water-consumptions.

### Reagents

cSBL was isolated using sequential chromatography with Sephadex G75, DEAE-cellulose, hydroxyapatite and SP-Sepharose, as previously described [24]. Pemetrexed disodium heptahydrate was purchased from LC Laboratories (Woburn, MA, USA). The caspase-3 and caspase-8 antibodies were purchased from Cell Signaling Technology, Inc. (Danvers, MA, USA). The caspase-9 antibody was purchased from Medical & Biological Laboratories Co., Ltd. (MBL; Nagoya, Japan). The β-actin antibody was obtained from Sigma-Aldrich (Merck KGaA, Darmstadt, Germany) and a horseradish peroxidase (HRP)-conjugated anti-mouse IgG antibody was purchased from Zymed Laboratories (Thermo Fisher Scientific, Inc., Waltham, MA, USA). An HRP-conjugated anti-rabbit IgG antibody was purchased from Cedarlane Laboratories (Burlington, Ontario, Canada).

### Annexin V staining assay

To investigate the induction of apoptosis, we evaluated Annexin V binding using an MEBCYTO apoptosis kit (MBL, Nagoya, Japan) according to the manufacturer’s instructions. Cells (5×10^4^ cells/mL) were cultured in 6-well plates (2 mL/well) and treated with cSBL (H2452: 1 μM; MSTO: 0.4 μM) for 24–72 h at 37°C in an atmosphere of 95% air and 5% CO_2_. Fluorescence intensity was subsequently detected using a FACSCalibur™ flow cytometer, and the data was analyzed using CELLQuest™ software version 6.0 (BD Biosciences, Franklin Lakes, NJ, USA).

### Detection of nuclear fragmentation

Cells (5×10^4^ cells/mL) cultured in a Cell Carrier-96 Ultra Microplate (100 μL/well) were treated with cSBL (H2452: 5 μM; MSTO: 2 μM) for 6, 24, 48 and 72 h, in triplicate. Then, cells were stained with 2 μg/mL Hoechst 33342 (Dojindo Laboratories, Kumamoto, Japan) for 1 h. The resulting images were acquired with the High-Content Analysis System Operetta CLS™ with NA 20X or 40X objectives, and the fragmentation index was calculated using Harmony™ Imaging and Analysis Software 4.6 (PerkinElmer Japan Co., Ltd., Kanagawa, Japan).

### Detection of caspase activity

The protein expression levels of activated caspase-3, -8, and -9 were analyzed using western blot assays. Cells (1×10^5^ cells/mL) cultured in 6-well plates (2 mL/well) were treated with cSBL (H2452: 5 μM; MSTO: 2 μM) for 1, 3, 6, 24, 48, and 72 h. Whole cell lysates were prepared using extraction buffer [150 mM NaCl, 10 mM Tris-HCl (pH 7.4), 5 mM EDTA, 1% Nonidet P-40, 0.1% sodium deoxycholate, and 0.1% sodium dodecyl sulfate] supplemented with cOmplete™ Mini EDTA-free Protease Inhibitor Cocktail tablets and PhosSTOP phosphatase inhibitor tablets (each 1 tablet/10 mL; Roche Applied Science, Indianapolis, IN, USA). Soluble proteins were collected, and the protein concentration was measured using a BCA protein assay kit (Thermo Fisher Scientific, Inc.) according to the manufacturer’s instructions. The proteins were separated using 10 or 14% SDS-PAGE and transferred onto Immobilon-P transfer membranes (EMD Millipore, Billerica, MA, USA). The membranes were sequentially incubated with primary and secondary antibodies diluted in Can Get Signal^®^ solution (Toyobo Co, Ltd., Osaka, Japan). The protein bands were detected using ECL Prime Western Blotting Detection Reagent (GE Healthcare, Little Chalfont, USA) or Chemi-Lumi One Super (Nacalai Tesque Inc., Kyoto, Japan).

Caspase enzymatic activity was measured using a Cell Meter™ Multiplexing Caspase-3/7, -8 and -9 Activity Assay Kit (AAT Bioquest, Inc., Sunnyvale, CA). Cells (5×10^4^ cells/mL) cultured in black 96-well plates (100 μL/well) were treated with cSBL (H2452: 5 μM; MSTO: 2 μM) for 1, 3, 6, 24, 48, and 72 h in triplicate. Substrate solution (100 μL) was added to each well, and the contents of the wells were mixed using a plate shaker for 30 sec. The cells were incubated at 37°C in a 5% CO_2_ atmosphere for 1 h. The luminescence in each well was measured using Infinite™ 200 PRO and i-control™ software (Tecan Japan Co., Ltd., Kanagawa, Japan).

### *In vivo* experiment

**Fig 1:**
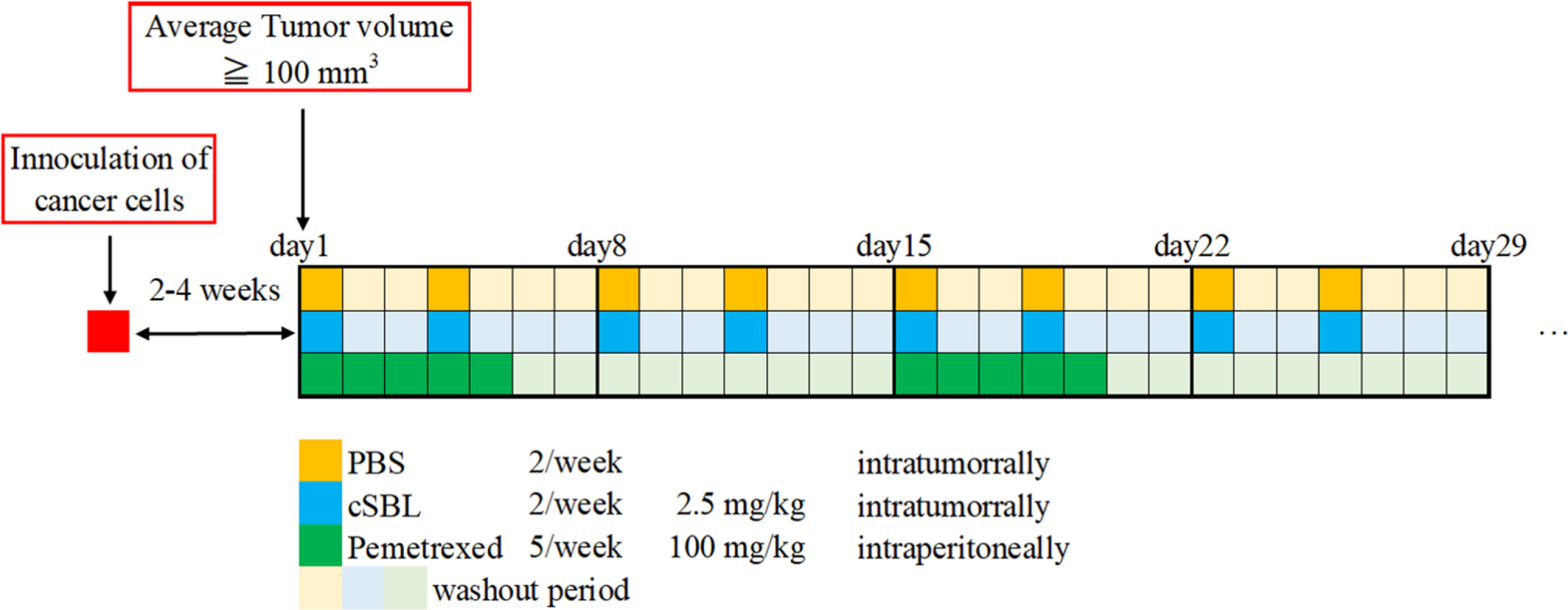
A schematic overview of the cancer cell injection and drug administration schedule. The cell suspension (100 μL/mouse, H2452; 5×10^6^ cells, MSTO; 2×10^6^ cells) was injected subcutaneously into the lower backs of mice. After 2–4 weeks, mice bearing tumors of 100–150 mm^3^ were randomly divided into 3 groups with 10 mice per group. Group 1 was administered PBS as control. Group 2 was injected intraperitoneally with pemetrexed (100 mg/kg) dissolved in sterile PBS on days 1–5 and 15–19. Group 3 was injected intratumorally with cSBL (2.5 mg/kg) twice per week for 4 weeks. Body weights and tumor sizes were measured twice per week. The endpoint of experiment was when the tumor diameter exceeded 200 mm^3^.

H2452 (5×10^6^ cells) and MSTO (2×10^6^ cells) cells were mixed with an equal volume of ice-cooled Corning™ Matrigel™ Basement Membrane Matrix (Corning, NY, USA), and an aliquot (100 μL) of suspended cells was subcutaneously injected into the lower backs of the mice. 2–4 weeks after inoculation (day 1), mice bearing tumors of 100–150 mm^3^ in volume were randomly divided into three groups, with 10 mice in each group. Group 1 was injected with 1 mL/kg vehicle (PBS) as the control. Group 2 was daily injected intraperitoneally with 100 mg/kg pemetrexed dissolved in sterile PBS on days 1–5 and 15–19. The method of administration the dosage of pemetrexed selected were based on the previously reported maximum tolerated dosage [36,37]. Group 3 was injected intratumorally with 2.5 mg/kg cSBL, twice weekly for four weeks. Body weights and tumor sizes were measured twice weekly. Solid tumor volumes were calculated as follows: 0.4×A×B^2^, with A and B representing the long and short tumor diameters (measured in mm), respectively. Tumor growth and body weight changes were evaluated as the ratio of each value against the baseline (day 1). Fig 1 shows the administration schedule used in the experiments. Mice were sacrificed by neck dislocation under ether anesthesia when tumor volumes reached 200 mm^3^.

### Drug combination studies

The effect of combination treatment on cell viability was determined using a WST-8 assay. Cells (5×10^4^ cells/mL) were cultured in 96-well plates (100 μL/well). The concentration of pemetrexed, cisplatin, or cSBL was based on the IC_50_ values obtained in the single-treatment experiments conducted in our prior study [35]. After 72 h, the cells were incubated with Cell Count Reagent SF (Nacalai Tesque Inc., Kyoto, Japan) at 37°C in a 5% CO_2_ atmosphere for 1–4 h. The absorbance of the resulting product at 450 nm was measured, and the background absorbance at 650 nm was subtracted. Combination Index (CI) and Dose Reduction Index (DRI) values were calculated using CompuSyn software (ComboSyn, Inc., Paramus, NJ), as described by Chou *et al* [38]. The experiments were conducted in triplicate. CI = 1 indicated an additive effect; CI<1 indicated a synergistic effect; CI>1 indicated an antagonistic effect. DRI = 1 indicated no dose reduction, whereas DRI>1 and <1 indicated favorable and unfavorable dose reductions, respectively.

### Statistical analysis

The results from ≥3 independent experiments, each performed in triplicate, are expressed as the mean ± standard deviation. Statistical analyses were conducted using GraphPad Prism 5.0, and comparisons were made using one-way analysis of variance (ANOVA) or two-way ANOVA followed by Bonferroni’s post hoc test. A P-value of <0.05 was considered statistically significant.

## Results

### cSBL induces apoptosis to H2452 and MSTO cells

**Fig 2:**
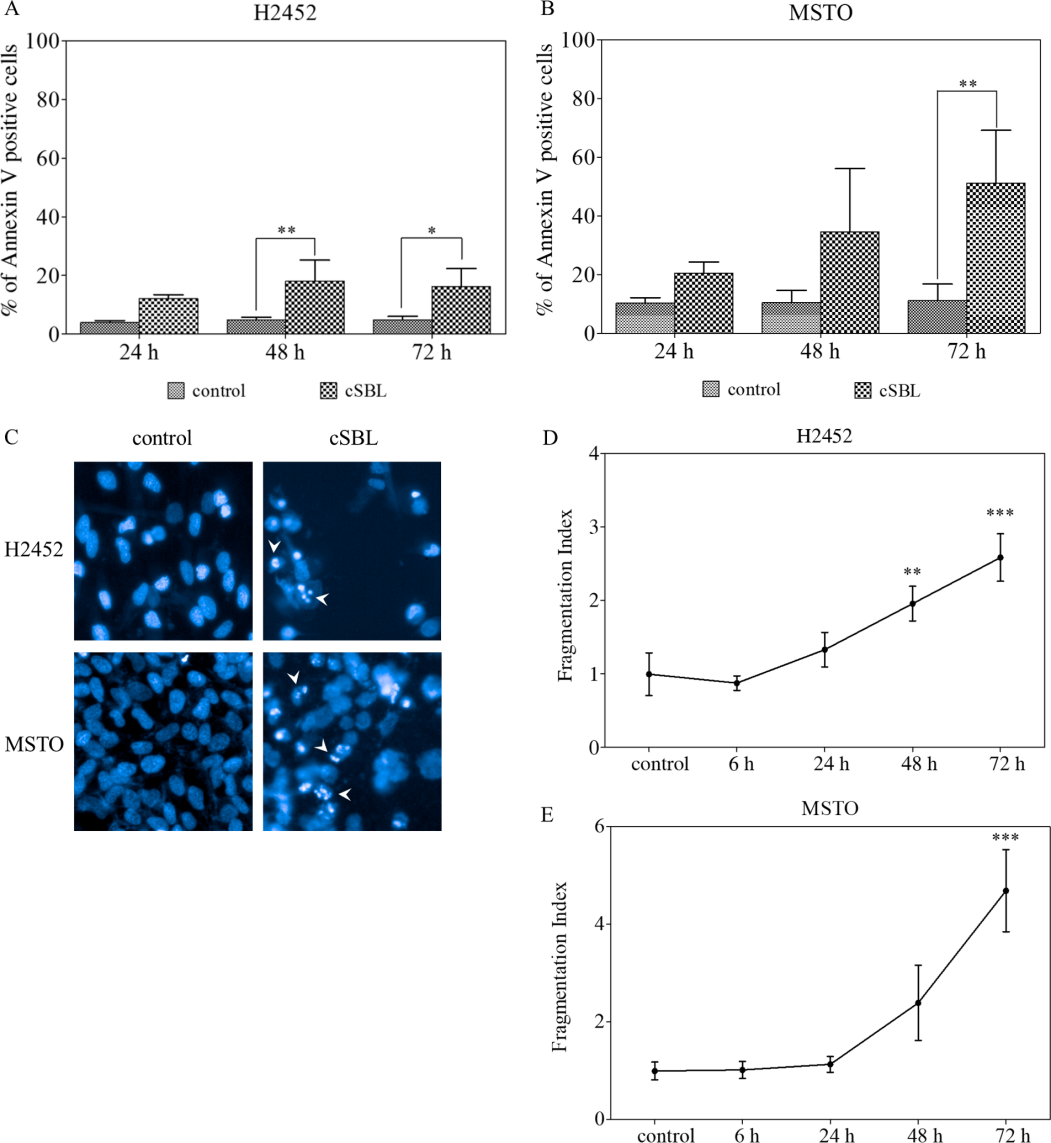
cSBL induced apoptotic changes in H2452 and MSTO cells. Cells were treated with cSBL for the indicated times. (A, B) Rate of apoptosis as indicated by the percentage of annexin V-positive cells. (C) Nuclear fragmentation images were captured using 40X objective; a false-colored image of the nuclei (blue) is shown. White arrowheads indicate the cells with condensed or fragmented nuclei. (D, E) Fragmentation index indicating the degree of nuclear fragmentation; a higher index indicates greater occurrence of fragmentation, calculated using the High-Content Analysis System. All data are expressed as the mean ± SD of three independent experiments. The statistical significance of these experiments compared with the control is shown as follows: *P<0.05, **P<0.01, ***P<0.001.

In order to investigate the antitumor activity of cSBL on xenograft-competent malignant mesothelioma cells, H2452 and MSTO cells were treated with cSBL and the antitumor mechanisms were analyzed. The percentage of Annexin V-positive cells was significantly increased in both H2452 (16.13%, 72 h) and MSTO (40.05%, 72 h) cells (Fig 2A and 2B). In addition, chromatin condensation and nuclear collapse were observed in the two cell types treated with cSBL (Fig 2C). Alterations to nuclear morphology were detected by High-Content Analysis Systems and numerically output as fragmentation indexes. As shown in Fig 2D and 2E, cSBL provoked significant nuclear morphology changes in time-dependent manner.

### cSBL-induced apoptosis is mediated by the intrinsic pathway

**Fig 3:**
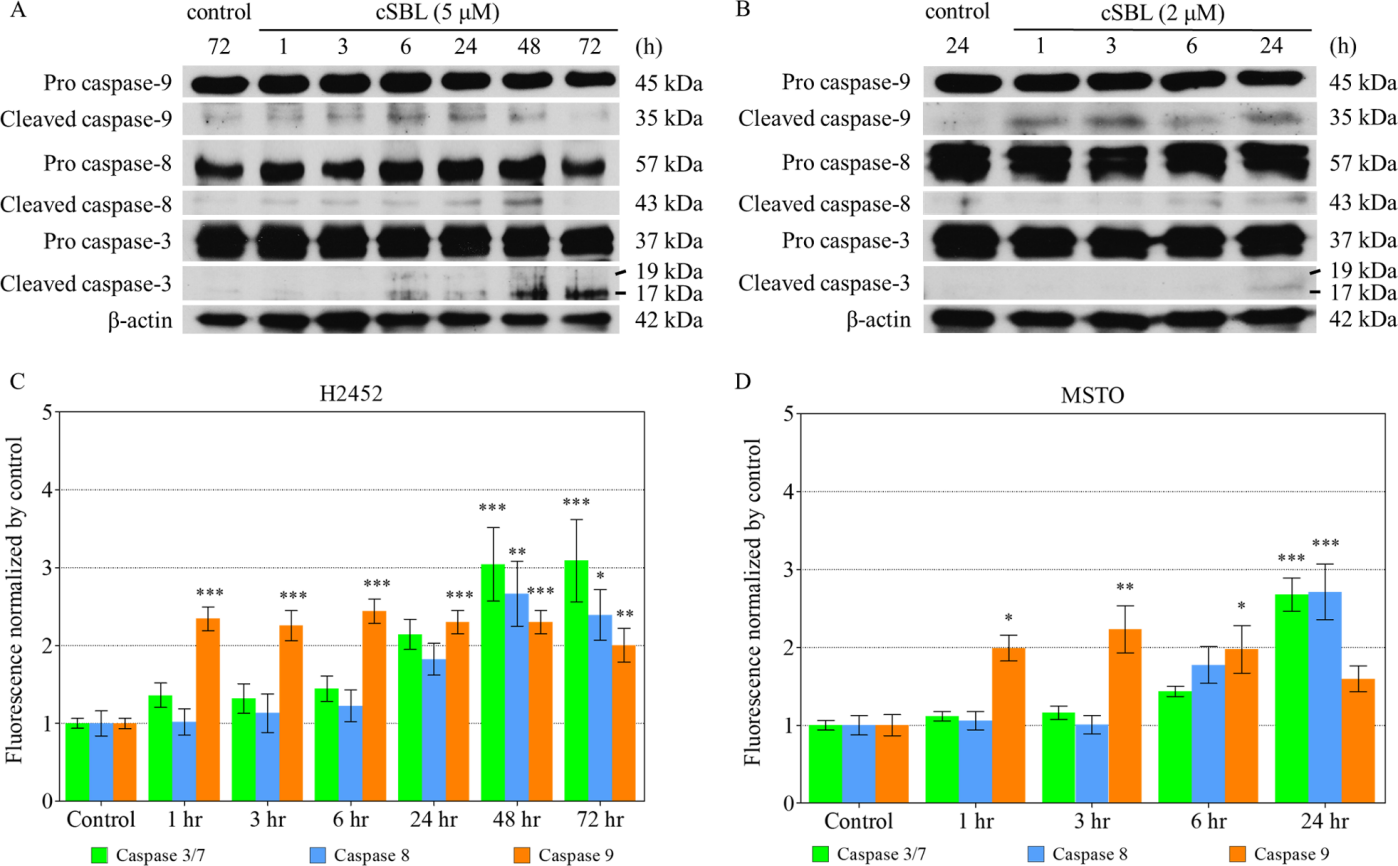
cSBL induced apoptosis in H2452 and MSTO cells via activation of the caspase pathway. Caspase-3, -8, and -9 activation was detected by western blotting (A, B) or fluorometry (C, D). Fluorometry was performed independently three times and data are expressed as the mean ± SD. The statistical significance of these experiments compared with the control is shown in as follows: *P<0.05, **P<0.01, ***P<0.001.

To obtain further insight into the mechanisms of cSBL-induced apoptosis in H2452 and MSTO cells, the activation of three key caspases was analyzed chronologically. The expression levels of activated caspase-9, -8 and -3 were detected by western blotting, and the substantial enzymatic activities of these caspases were evaluated by fluorometric analysis. As shown in Fig 3A and 3B, increased levels of activated caspase-9 were observed from 6 h and 1 h in H2452 and MSTO cells, respectively. After that, activated caspase-8 began to be observed from 24 h in H2452 and 6 h in MSTO cells. The appearance of activated caspase-3 was recorded from 48 h in H2452 and 24 h in MSTO cells. Consistently, the enzymatic activity of caspase-9 was significantly enhanced from 1 h in H2452 and MSTO cells, and the levels of caspase-8 and -3/7 increased almost simultaneously (H2452: 48 h; MSTO: 24 h; Fig 3C and 3D). Thus, caspase-9 was activated prior to caspase-8 and -3, indicating that the intrinsic apoptotic pathway was involved.

### cSBL inhibits cancer cell proliferation without inducing weight loss in xenograft models

**Fig 4:**
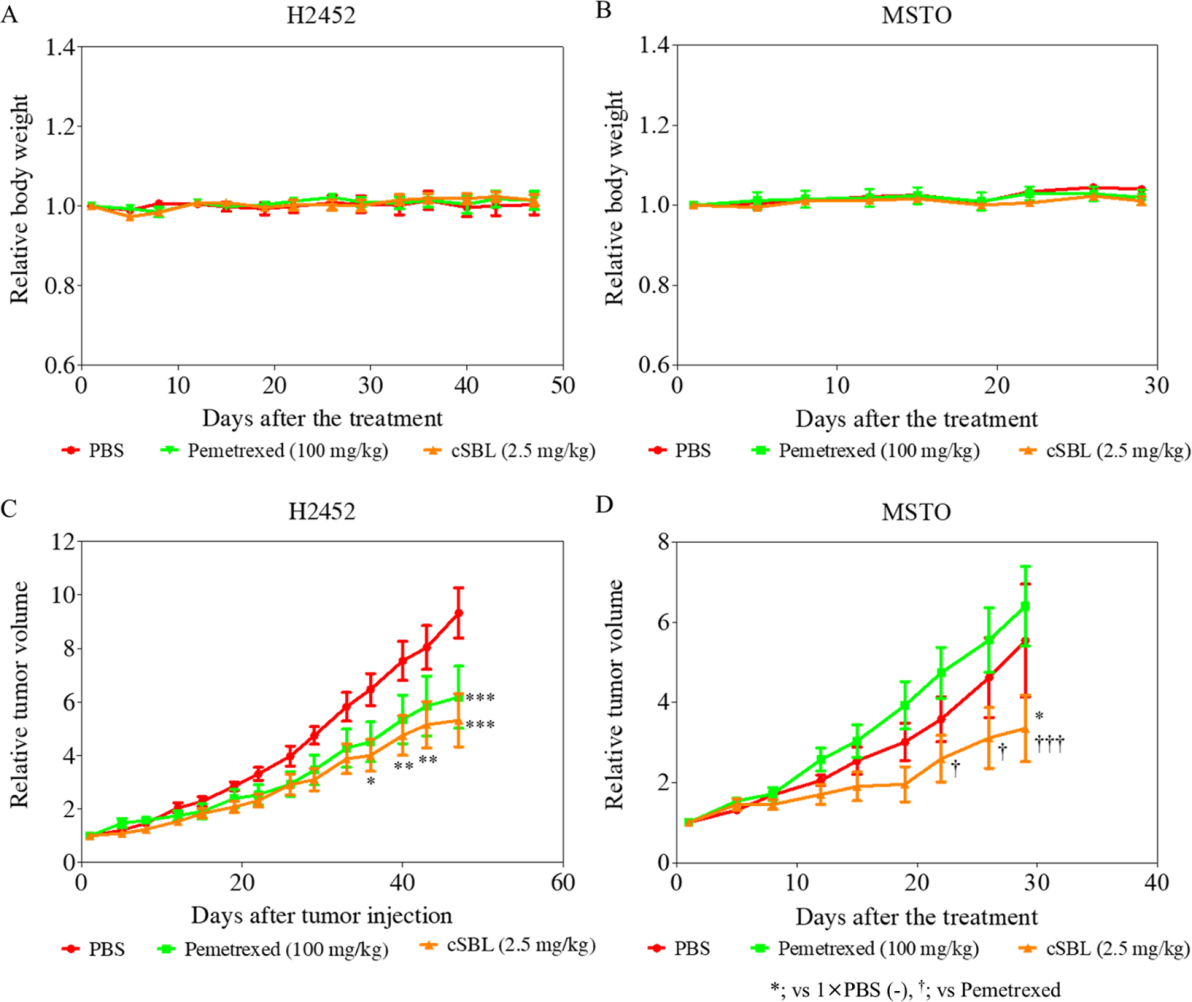
cSBL showed *in vivo* cytotoxicity without inducing loss of body weight. Mice were randomly divided into 3 groups with 10 mice in each group. Groups 1, 2, and 3 were injected PBS, pemetrexed (100 mg/kg, intraperitoneally), and cSBL (2.5 mg/kg, intratumorally), respectively. Body weights and tumor sizes were measured twice per week. Tumor volumes were calculated as follows: 0.4×A×B^2^, where A and B represent the long and short diameters (in mm) of the tumor, respectively. Relative body weight (A, B) and relative tumor volume (C, D) are plotted as the mean of each group ± SD at each timepoint. The statistical significance of these experiments compared with PBS (*) or pemetrexed (^†^) is shown as follows: *P<0.05, **P<0.01, ***P<0.001, ^†^P<0.01, ^†††^P<0.001.

To examine the effects of cSBL on tumor growth *in vivo*, nude mice were inoculated with H2452 and MSTO cells. cSBL was administered intratumorally and the effects of pemetrexed were also assessed according to previously reported experimental conditions [36,37] (Fig 1). The rate of changes to animal weights and tumor volumes were monitored following the administration of each agent. As shown in Fig 4A and 4B, body weight changes were not observed in any of the groups. In the H2452 xenograft model, cSBL and pemetrexed each significantly inhibited tumor growth compared with the PBS group (P<0.05), and the cSBL group showed a growth inhibition effect earlier (after 36 days of treatment) than that of the pemetrexed group (after 47 days of treatment) (Fig 4C). Conversely, for the MSTO xenograft group, significant inhibition of cancer growth was observed only in the cSBL-treated group after 29 days of treatment (P<0.05; Fig 4D).

### cSBL and pemetrexed exhibit a strong synergistic effect in H2452 and MSTO cells

**Fig 5:**
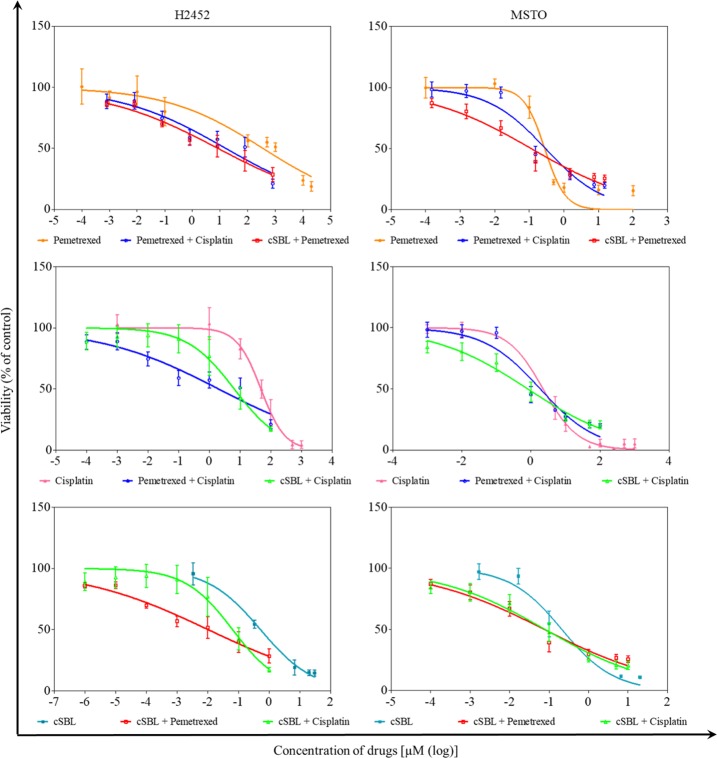
Viability curves of H2452 and MSTO cells treated with pemetrexed, cisplatin, and cSBL, either alone or in combination. Each group of cells was treated with fixed concentration ratios of pemetrexed:cisplatin:cSBL as follows: 800:100:1 (for H2452 cells) or 3:20:2 (for MSTO cells). Each data point represents the mean ± SD of at least three independent WST-8 assays. Each sample was plated in triplicate. The y-axis indicates the viability of cells. The x-axis indicates the concentration of pemetrexed (upper), cisplatin (middle), or cSBL (lower).

**Fig 6:**
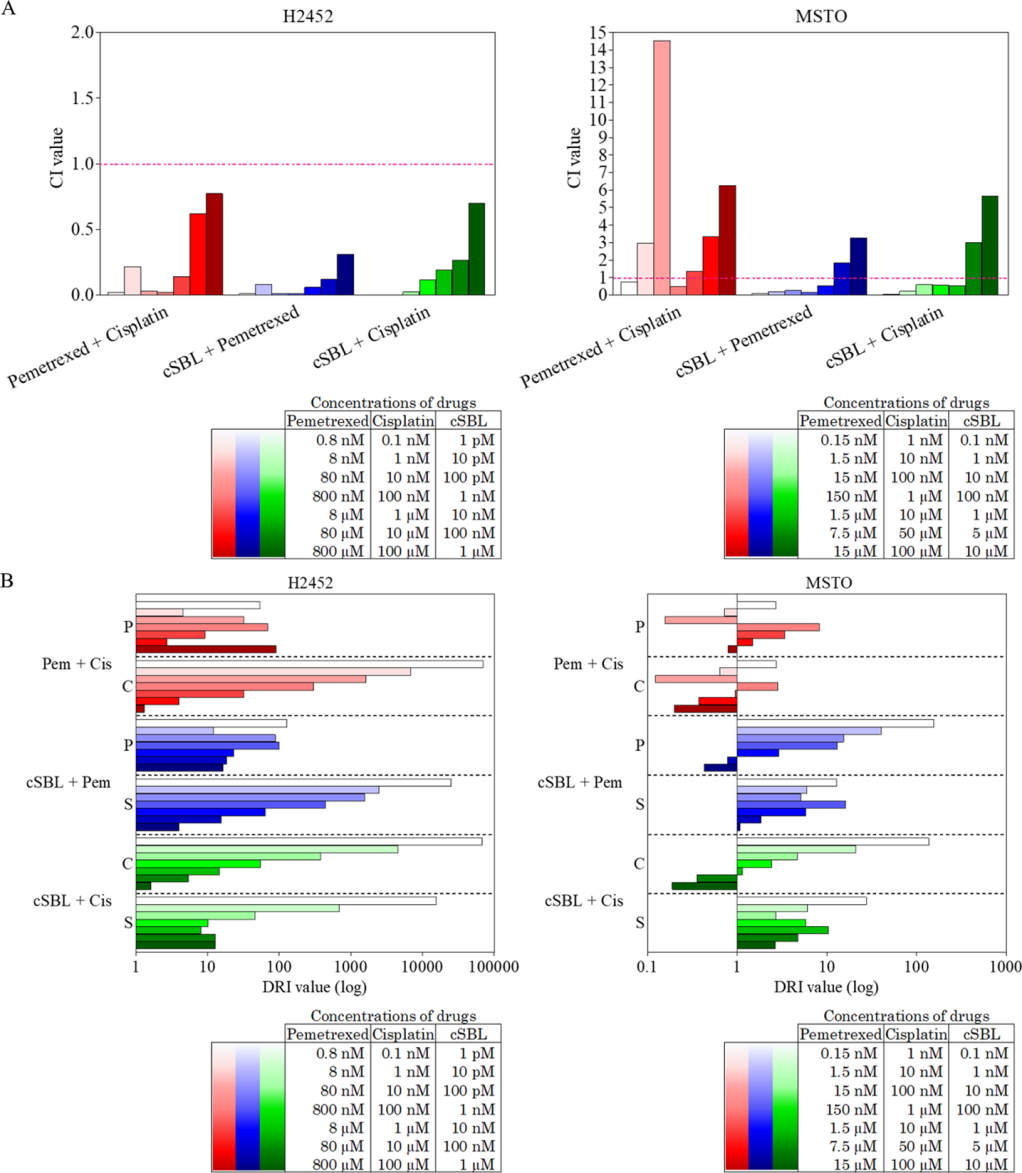
Pharmacological interactions between pemetrexed, cisplatin and cSBL in H2452 and MSTO cells. (A) CI values of each combination (CI = 1 indicates an additive effect; CI<1 indicates a synergistic effect; and CI>1 indicates an antagonistic effect). (B) DRI values of each reagent (DRI = 1 indicates no dose reduction; whereas DRI >1 and <1 indicate favorable and unfavorable dose-reductions, respectively). Pem or P, pemetrexed; Cis or C, cisplatin; S, cSBL.

Finally, we performed the *in vitro* combination study of cSBL with other reagents. In addition to pemetrexed, cisplatin, an existing drug for malignant mesothelioma usually used in combination with pemetrexed, was chosen for the test reagent. Pharmacological interactions between these three agents were investigated by evaluating the viability of H2452 and MSTO cells treated with pemetrexed + cisplatin, cSBL + pemetrexed, and cSBL + cisplatin. The drug concentration in each combination regimen was based on the IC_50_ value for each agent previously determined via single treatments [35]. The viability curves for each drug in single or combination treatments are presented in Fig 5. In H2452 cells, all combinations decreased cell viability to a greater extent than each single treatment over the whole concentration range. Similar tendencies were observed in MSTO cells over a wide concentration range, although the combination effects appeared to saturate at a higher concentration. To evaluate the synergistic effect of each drug combination, CI values were calculated. At each experimental concentration (Fig 6A) in H2452 cells, the CI values for all combinations were <1, indicating that all combinations were synergistic. cSBL + pemetrexed showed the highest synergistic effect at all concentration points. In MSTO cells, CI values at the highest two concentration points of all combinations exhibited antagonism rather than synergism; however, cSBL-containing combinations (cSBL + pemetrexed; cSBL + cisplatin) exhibited high synergism in the mid-low concentration range. The pemetrexed + cisplatin combination in MSTO cells showed dispersion and high CI values at various concentrations. Furthermore, we calculated DRI values, representing the index of the fold-number that each drug combination dose could be reduced by compared with that of each drug alone (Fig 6B). In H2452 cells, all combinations had high DRI values. In MSTO cells, high DRI values were observed in the cSBL-containing combinations, particularly in the low concentration range. However, the pemetrexed + cisplatin combination had low DRI values at all concentrations.

## Discussion

We previously demonstrated that cSBL induces apoptosis in H28 (sarcomatoid histological type), MESO-1 and MESO-4 (epithelioid type) cells, but not in normal Met5A mesothelial cells, by detecting elevated proportions of Annexin V positive cells following cSBL treatment [34]. Furthermore, from the investigations in which H2452 (epithelioid type) and MSTO (biphasic type) were utilized, in addition to aforementioned cell lines, higher cancer-selectivity of cSBL was observed compared with either pemetrexed or cisplatin in their antiproliferative effects [35]. The antitumor mechanism of cSBL in malignant mesothelioma has been well-documented in H28 cells; it was revealed that cSBL treatment activates the caspase cascade, the proapoptotic Bcl-2 family proteins Bik and Bim, as well as JNK and p38 MAPKs, consequently inducing apoptosis in these cells. However, although the effectiveness of cSBL against mesothelioma *in vitro* has been reported, the *in vivo* efficacy of cSBL has not been investigated to date.

Although H28, MESO-1, and MESO-4 cells did not show tumorigenicity in the nude mice used, we succeeded in establishing malignant mesothelioma xenografts with H2452 and MSTO cells. First, the antitumor effects of cSBL on these two cell lines were investigated *in vitro*. cSBL induced typical apoptotic changes, such as phosphatidylserine externalization, nuclear condensation and fragmentation, in both cells in a time-dependent manner (Fig 2). Moreover, caspase-9 was activated by cSBL treatment earlier and more strongly than caspase-8, indicating that apoptosis was induced through the intrinsic pathway (Fig 3). In the *in vivo* studies, no obvious toxicities or body weight changes were observed during the experimental period in any group (Fig 4A and 4B). In both types of xenograft, significant tumor growth suppression was observed in cSBL-treated groups compared with control groups. In H2452 xenografted groups, cSBL showed a tumor-suppressive effect earlier than that of the pemetrexed-treated group, and the antitumor effect of pemetrexed was not observed in the MSTO xenografts (Fig 4C and 4D). The reason for the lack of effect by pemetrexed is uncertain; we speculate that the high growth rate of MSTO cells in the xenograft model may contribute to this phenomenon. We were unable to compare the effects of cSBL and pemetrexed directly due to the differences in the dosing conditions; however, our observations indicate that cSBL could potentially inhibit the tumor growth of mesothelioma without any toxicity, even if previously established pemetrexed administration had little or no effect. From these results, it was suggested that cSBL had the capability to inhibit tumor growth in xenografted mice. Thus, cSBL may be safely used, and further studies are required to determine the maximal tolerated dose of cSBL in order to optimize its efficacy.

Combination therapy, a treatment modality that combines two or more therapeutic agents to reduce the risk of drug resistance or adverse effects while simultaneously providing therapeutic anti-cancer benefits, is a mainstay of current cancer therapy [39]. In fact, combination treatments comprising pemetrexed and cisplatin are used for the treatment of mesothelioma as a standard regimen. We previously demonstrated that the cSBL + pemetrexed combination exerted stronger cytotoxicity and synergism compared with the pemetrexed + cisplatin combination in H28 cell lines. The cytostatic effect of pemetrexed and the cytotoxic effect of cSBL cooperated without any repulsion, although the effects of pemetrexed and cisplatin on cyclin A expression were counteractive when used in combination [35]. In the present study, we evaluated the generality of the prominent synergistic effect of the cSBL + pemetrexed combination, utilizing H2452 and MSTO cells, by calculating CI and DRI values. The cSBL + pemetrexed combination exhibited the highest synergism of the three combinations tested in both cell lines (Fig 5B). Surprisingly, in MSTO cells, the pemetrexed + cisplatin combination appeared to be antagonistic rather than synergistic or additive at the most of concentration points tested. High DRI values (Fig 5C) were calculated for all combinations, except for pemetrexed + cisplatin in MSTO cells. These results suggest that cSBL + pemetrexed may be a rational treatment combination for several types of malignant mesothelioma. On the other hand, the current gold-standard regimen for malignant mesothelioma, pemetrexed + cisplatin, may be ineffective, depending on the cell type, with respect to synergism (i.e., undesired adverse effects may easily occur in some circumstances, depending on the patient).

Although the combination of pemetrexed and cisplatin has been demonstrated to prolong the survival of patients with malignant mesothelioma, the median survival is only 12 months, and the response rate is ~40% [12]. Thus, almost half of all mesothelioma patients are initially resistant, and all eventually develop resistance [40]. Therefore, researches to improve the malignant mesothelioma therapy have been actively attempt. The combinations of carboplatin and pemetrexed, or gemcitabine and cisplatin showed comparable outcomes with pemetrexed and cisplatin combination in phase- II trials [41–43]. The French Mesothelioma Avastin Cisplatin Pemetrexed Study (MAPS) demonstrated a statistically significant improvement in the median overall survival time using a combination of cisplatin, pemetrexed and bevacizumab, a monoclonal antibody that binds VEGF and blocks its interaction with the VEGF receptor [44]. In addition, several other molecular targeting and immunotherapeutic agents, such as anti-EGFR signaling agent and anti-programmed cell death 1 (PD-1) antibodies, are currently being investigated in clinical trials [43]. In this study, cSBL was demonstrated to induce apoptosis and inhibit tumor cell growth in xenografted mice. CI analysis also evidenced a prominent combinatory effect of cSBL with pemetrexed. As cSBL is a novel candidate anti-cancer agent that exerts antitumor activity through targeting RNA (which represents a novel class of potential therapeutic targets), it may provide a new option for the chemotherapeutic treatment of malignant mesothelioma. This is particularly true among patients with pemetrexed resistance, as cSBL was effective in pemetrexed-resistant cells, as shown in the current study and in previous report [35].

## Conclusions

cSBL induced apoptosis in H2452 and MSTO cells via the intrinsic apoptotic pathway. *In vivo*, cSBL treatment inhibited tumor growth in multiple xenograft models without any undesirable adverse effects. A higher efficacy was achieved by the use of cSBL + pemetrexed in mesothelioma cells compared with pemetrexed + cisplatin. To the best of our knowledge, this is the first report to demonstrate the antitumor efficacy of cSBL in human malignant mesothelioma xenograft models. cSBL has potential as a novel treatment for malignant mesothelioma.
